# Supplementary material for: A lipoprotein/β-barrel complex monitors lipopolysaccharide integrity transducing information across the outer membrane
Source: eLife. 2016 Jun 10;5:e15276. doi: 10.7554/eLife.15276 (PMC4942254; doi:10.7554/eLife.15276)
Supplement: Supplementary file 1. — DOI: http://dx.doi.org/10.7554/eLife.15276.013 [file elife-15276-supp1.docx]

**Strains used in this study**

| MC4100 | F-ჼ*araD139* (*argF-lac*)*U169* *rpsL150 relA1 flb5301 deoC1 ptsF25 thi* | (Silhavy et al. 1984) |
| --- | --- | --- |
| DH300 | *PrprA-lacZ* | (Majdalani et al. 2002) |
| JCM 158 | MC4100 *araR*/-(parent for all strains) | (Malinverni et al. 2006) |
| MG1029 | Δl*ptE2*::kan / p*lpt*E(R91D,K136D) | (Malojcic et al. 2014) |
| AK-265 | *PrprA-lacZ* |  |
| AK-266 | *ΔrcsF PrprA-lacZ* |  |
| AK-638 | *ΔwaaP PrprA-lacZ* |  |
| AK-430 | *ompA*::Kan *PrprA-lacZ* |  |
| AK-627 | *bamE::*Cm *PrprA-lacZ* |  |
| AK-628 | *ΔwaaP bamE*::Cm *PrprA-lacZ* |  |
| AK-645 | *ΔwaaP ompA*::Kan *PrprA-lacZ* |  |
| AK-653 | *phoP*::Kan *PrprA-lacZ* |  |
| AK-654 | *ΔwaaP phoP*::Kan *PrprA-lacZ* |  |
| AK-663 | *ΔrcsF* pZS21::*rcsF*_WT *PrprA-lacZ* |  |
| AK-680 | *ΔrcsF* pZS21 *PrprA-lacZ* |  |
| AK-681 | *ΔrcsF* pZS21:: *rcsF_A55Y* *PrprA-lacZ* |  |
| AK-664 | *ΔrcsF* pZS21::*rcsF_A5* *PrprA-lacZ* |  |
| AK-671 | *ΔrcsF ΔwaaP* *PrprA-lacZ* |  |
| AK-672 | *ΔrcsF ΔwaaP* pZS21 *PrprA-lacZ* |  |
| AK-673 | *ΔrcsF ΔwaaP* pZS21::*rcsF*_WT *PrprA-lacZ* |  |
| AK-684 | *ΔrcsF ΔwaaP* pZS21:: *rcsF_A55Y* *PrprA-lacZ* |  |
| AK-674 | *ΔrcsF ΔwaaP* pZS21::*rcsF_A5* *PrprA-lacZ* |  |
| AK-770 | *waaC::Kan PrprA-lacZ* |  |
| AK-434 | *waaP::Kan PrprA-lacZ* |  |
| AK-771 | *waaF::Kan PrprA-lacZ* |  |
| AK-772 | *waaQ::Kan PrprA-lacZ* |  |
| AK-773 | *waaY::Kan PrprA-lacZ* |  |
| AK-774 | *waaG::Kan PrprA-lacZ* |  |
| AK-775 | *waaB::Kan PrprA-lacZ* |  |
| AK-776 | *waaO::Kan PrprA-lacZ* |  |
| AK-777 | *waaJ::Kan PrprA-lacZ* |  |
| AK-778 | *waaL::Kan PrprA-lacZ* |  |
| AK-779 | *ΔpldA mlaA::*kan *nadA::Tn10 PrprA-lacZ* |  |
| AK-780 | *lptD4213 nadA::*Tn10 *PrprA-lacZ* |  |
| AK-781 | *ΔlptE / plptE(R91D,K136D) nadA::Tn10 PrprA-lacZ* |  |
| AK-871 | *ΔpldA ΔmlaA waaP::kan nadA::Tn10 PrprA-lacZ* |  |
| AK-873 | *lptD4213 waaP::kan nadA::Tn10 PrprA-lacZ* |  |
| AK-874­­ | *ΔlptE / plptE(R91D,K136D) waaP::kan nadA::Tn10 PrprA-lacZ* |  |
